# Overview of nursing ethics teaching in Brazilian public higher education institutions

**DOI:** 10.1590/0034-7167-2022-0808

**Published:** 2023-12-04

**Authors:** Alexandre de Assis Bueno, Renata Alessandra Evangelista, Tassiana Potrich, Luana Prado Figueredo, Cristiane Costa Reis da Silva, Gilberto Tadeu Reis da Silva, Marta Manzano-Garcia, Blanca Espina Jerez

**Affiliations:** I Universidade Federal de Catalão. Catalão, Goiás, Brazil; II Universidade Federal da Fronteira Sul. Chapecó, Santa Catarina, Brazil; III Universidade Santo Amaro. São Paulo, São Paulo, Brazil; IV Universidade Federal do Amazonas. Coari, Amazonas, Brazil; V Universidade Federal da Bahia. Salvador, Bahia, Brazil; VI Gerencia Regional de Salud de Castilla y León. Salamanca, Castillas Leon, Spain; VII University of Alicante. Alicante, Comunidade Valenciana, Spain

**Keywords:** Nursing, Ethics, Ethics, Nursing, Students, Nursing, Higher Education, Enfermería, Ética, Ética en Enfermería, Estudiantes de Enfermería, Enseñanza Superior, Enfermagem, Ética, Ética em Enfermagem, Estudantes de Enfermagem, Ensino Superior

## Abstract

**Objectives:**

to outline the teaching of ethics in undergraduate Nursing programs in Brazilian public higher education institutions.

**Methods:**

descriptive and exploratory study, carried out through the documentary analysis of pedagogical projects of undergraduate Nursing programs in Brazil.

**Results:**

153 active undergraduate Nursing programs were found, of which 106 provide the pedagogical project. In addition to deontological teaching, the teaching of ethics was identified in a transversal way associated with themes such as Social Context, Hospital and Community Care, Pharmacology, Systematization of Nursing Care, Surgical Nursing, Epidemiology, Palliative Care, Management in Nursing, Diversity, Women’s, Children’s, Adolescent’s, Adult’s and Older People’s Health, and Mental Health.

**Final Considerations:**

the challenge in teaching nursing ethics is its integration with each action of caring, teaching and managing.

## RESUMO

**Objetivos:**

traçar o panorama do ensino da ética nos cursos de graduação em Enfermagem nas instituições públicas de ensino superior brasileiras.

**Métodos:**

estudo descritivo e exploratório, realizado por meio da análise documental de projetos pedagógicos de cursos de graduação em Enfermagem no Brasil.

**Resultados:**

foram encontrados 153 cursos ativos de graduação em Enfermagem, dos quais 106 disponibilizam o projeto pedagógico. Além do ensino deontológico, foi identificado o ensino de ética de forma transversal associada a temas como Contexto Social, Atenção Hospitalar e Comunitária, Farmacologia, Sistematização da Assistência em Enfermagem, Enfermagem Cirúrgica, Epidemiologia, Cuidados Paliativos, Gestão em Enfermagem, Diversidade, Saúde da Mulher, Criança, Adolescente, Adulto e Idoso e Saúde Mental.

**Considerações Finais:**

o desafio no ensino da ética em enfermagem é sua integração com cada ação de cuidar, ensinar e gerenciar.

## RESUMEN

**Objetivos:**

trazar el panorama de la enseñanza de la ética en los cursos de graduación en Enfermería en las instituciones públicas de enseñanza superior brasileñas.

**Métodos:**

estudio descriptivo y exploratorio, realizado por medio del análisis documental de proyectos pedagógicos de cursos de graduación en Enfermería en Brasil.

**Resultados:**

han sido encontrados 153 cursos activos de graduación en Enfermería, de los cuales 106 tienen disponible el proyecto pedagógico. Además de la enseñanza deóntica, fue identificado la enseñanza de ética de forma transversal relacionada a temas como Contexto Social, Atención Hospitalaria y Comunitaria, Farmacología, Sistematización de la Asistencia en Enfermería, Enfermería Quirúrgica, Epidemiología, Cuidados Paliativos, Gestión en Enfermería, Diversidad, Salud de la Mujer, Niño, Adolescente, Adulto y Anciano y Salud Mental.

**Consideraciones Finales:**

el desafío en la enseñanza de la ética en enfermería y su integración con cada acción de cuidar, enseñar y administrar.

## INTRODUCTION

In its essence, nursing is understood as the development of comprehensive and multidimensional care actions for the person, family and community, in their different contexts and life circumstances; such actions are based on their own components of scientific and technical knowledge, with emphasis on training based on health, ethical, social and political precepts, which are processed by care, management, research and teaching^(1)^.

In nursing, when developing comprehensive care, it is essential to assume an attitude of respect and acceptance with regard to the values, beliefs and attitudes of the client/patient, in accordance with the set of moral principles, individual, professional and organizational rights and duties^(2-3)^. This set is linked to care and managerial competencies, in the relation of know-how, characterized by an interface of professional, organizational and social correlations that is structured in the fields of Nursing practice and characterizes it as a predominantly relational act^(1)^.

However, with advances in technology, the growing complexity of the health care system and contemporary issues associated with diversity and inclusion present nurses with recurring situations that raise ethical problems^(4)^.

Situations related to the covid-19 pandemic, organ transplantation, epidemics such as AIDS, teenage pregnancy, attempts at self-extermination, exposures to clients who do not have access to health care, health judicialization and organizational pressures to contain costs create complex situations for ethical decision-making in the use of available resources in view of the needs presented^(5)^.

In addition, nursing deals with historical issues that challenge its ethical pillars in strengthening the professional identity based on “intelligence, education and strength of character”^(6)^, namely medical protagonism in the health area, low remuneration, overload and long working hours^(6-7)^. These conditions interfere with the practice of daily nursing care, as they create noise in the transition from knowledge to doing so based on strong moral values. These characteristics remain crucial for nursing practice^(6-7)^.

Strength of character has been named as moral sensitivity and is currently perceived as central to the nurse’s orientation in the composition of patient-centered and humanized care. The teaching of ethics in Nursing curricula is seen as a pedagogical path for the development of this moral sensitivity with a direct impact on both the quality of learning and the professional identity of nurses^(5,8)^.

That said, it is necessary to rethink critical and reflective training not only with regard to technically skilled professionals, but above all with regard to professional ethics that has permeated nursing actions in such challenging circumstances and care scenarios, as in recent times^(3)^.

## OBJECTIVES

To outline the teaching of ethics in undergraduate Nursing programs in Brazilian public higher education institutions.

## METHODS

### Ethical Aspects

Ethical review and committee approval were waived for this study due to the use of data available on open access university websites. For the same reason, informed consent for this study is not applicable.

### Study design

Study of qualitative and descriptive approach through documentary analysis of pedagogical projects of undergraduate Nursing programs in Brazilian public institutions. This analysis can be defined by the review and evaluation of organizational and institutional documents as a source of data in qualitative research, and can be collected from various locations, such as public domain records, websites, personal documents and physical evidence^(9)^. The COREQ guidelines were followed (Consolidated criteria for Reporting Qualitative Research)^(10)^.

### Study period and location

The data search strategy used included the identification of nursing programs in public institutions of higher education in Brazil, through the platform Cadastro Nacional de Cursos e Instituições de Educação Superior - Cadastro e-MEC, of the Ministry of Education. Then, a visit was made to the institution’s website and, when necessary, to the nursing program to locate, access, review and evaluate the pedagogical project of the program, curriculum or other document that described the subjects offered by the program. This period ranged from July to September 2022.

### Population and sample

The population consisted of Brazilian undergraduate Nursing programs. The sample consisted of active undergraduate Nursing programs in the e-MEC system, with a pedagogical project of the program (PPP) or curriculum available on the websites of the universities or programs. To compose the results, the institutions of the three types of public segments were considered: municipal, state and federal.

### Inclusion Criteria

Public, face-to-face and active administrative institutions were included.

### Study protocol

The search for the pedagogical projects of the programs or curriculum was carried out by four researchers, independently and at the same time. To this end, a structured form was used, containing items consistent with the PPP: HEI region, category, degree, type of subject, semester/year, and workload. The data collection technique included the search for Nursing programs in public institutions of higher education in Brazil, through the platform Cadastro Nacional de Cursos e Instituições de Educação Superior - Cadastro e-MEC, of the Ministry of Education. Subsequently, the websites of the institutions and, when necessary, the Nursing program were consulted to locate, access and analyze, review and evaluate the pedagogical project of the program, curriculum or other document that described the Ethics disciplines offered by the program.

### Analysis of results and statistics

For lexical analysis of the contents present in the menus, the Reinert method was used, supported by the software Interface de R pour les Analyses Multidimensionnelles de Textes et de Questionnaires - IRaMuTeQ^®^. This method, which follows a top-down hierarchical analysis format, provides a number of classes and statistical clues in the form of typical words and text segments. Specifically, the software classifies text segments according to their respective vocabularies and subsequently groups them based on word frequency to obtain classes of terms similar to each other and the degree of connection of one class with the others^(11-12)^.

Reinert operations are statistical, transparent, and reproducible to the final stage of interpretation, where the analyst assigns a label to each specific vocabulary set that the software has identified as a lexical world based on co-occurrences and distribution patterns.

Finally, as a complementary analysis, IRaMuTeQ also performs a lexical similarity analysis. This analysis presents in graphical format the structure of a corpus, distinguishing between the shared parts and the specificities of the coded variables. This allows the link between the different shapes in the text segments to emerge. That is, this analysis allows detecting the co-occurrences of words, providing information on the connectivity of words and, thus, helping to identify the structure of the content of a textual corpus. It also makes it possible to locate the parts and specificities shared according to the descriptive variables found in the analysis^(11-13)^.

The units of meaning detected by the analysis of similarity and represented by the maximum tree were submitted to content analysis to extract thematic meanings or lexical signifiers, through the simplest elements of the text associated with the teaching of ethics in the training of nurses in public institutions of higher education^(14)^.

## RESULTS

Among the PPPs identified and analyzed, 21 were published before 2011; between 2012 and 2013, there were 15; in the years 2014 to 2016, there were 28; the period from 2017 to 2019 includes 33; and, finally, 9 are between the years 2020 and 2022 (Table 1).

**Table 1:** Specific disciplines of Ethics of Professional Practice, according to the type of program, subject and year of the program, Brazil, 2022

HEI	Degree		PPP available on the internet	Type of discipline			Program Year			
	Bac^^*^^ n (%)	Lic^^**^^ n (%)		Ob^^***^^ n (%)	Opt^^****^^ n (%)	NL^^*****^^ n (%)	1^st^ n (%)	2^nd^ n (%)	3^rd^ n (%)	4^th^ n (%)
Federal	80 (52.3)	3 (2.0)	55 (66.2%)	73 (46.7)	3 (1.9)	6 (3.8)	38 (24.3)	27 (17)	12 (7.7)	13 (8.3)
State	64 (41.8)	2 (1.3)	49 (74%)	61 (39.1)	2 (1.2)	4 (2.5)	28 (17.9)	15 (9.6)	7 (4.5)	9 (5.8)
Municipal	4 (2.6)	0	2 (50%)	7 (4.5)	0	0	4 (2.5)	1 (0.64)	0	2 (1.3)
Total	153 (100%)		106 (71.6%)	156 (100%)			156 (100%)			

HEI - higher education institutions; PPP - pedagogical project of the program;^*^Bachelor’s Degree;

** Licentiate Degree;

*** Mandatory;

**** Optional;

***** Free Core. Source: Pedagogical projects (PPP) of Nursing programs at public universities in Brazil.

* *Untranslated Image. Generated from analysis by software Interface de R pour les Analyses Multidimensionnelles de Textes et de Questionnaires - Iramuteq*

In Brazil, 153 nursing programs were found in public higher education institutions (HEIs). Of these, only 106 pedagogical projects were available on the Internet, in which the offer of 156 disciplines that address ethics was observed, 103 specific disciplines of ethics and 53 disciplines contemplating ethics as a topic in their syllabus.

As for the characterization of the subjects by segments of the HEIs, it is observed, in the pedagogical projects available on the websites, the offer of ethics in predominantly mandatory subjects, corresponding to 90.3% of the total offers, being offered mainly in the first two years of training (71.94%).

The 53 non-specific disciplines present ethics in a transversal way associated with themes such as Social Context, Hospital and Community Care, Pharmacology, Systematization of Nursing Care, Surgical Nursing, Epidemiology, Palliative Care, Nursing Management, Diversity, Health of Women, Child, Adolescent, Adult and Older People, and Mental Health.

This trend of transversality of ethics is distributed in 22 PPPs, 2 before 2011 and 20 after 2017, which indicates a contemporary perspective in the teaching of ethics, problematized in contexts inherent to nursing work in the care, investigative and managerial dimensions.

This aspect expands the traditional and predominant offer of ethics, in specific disciplines, in which there is a direct association with legislation and professional practice. Thus, ethics is evidenced in the historical process of construction of the professional identity of nursing with emphasis on its legal aspects that determine the rights and duties of nursing professionals in their professional practice.

**Figure 1:**
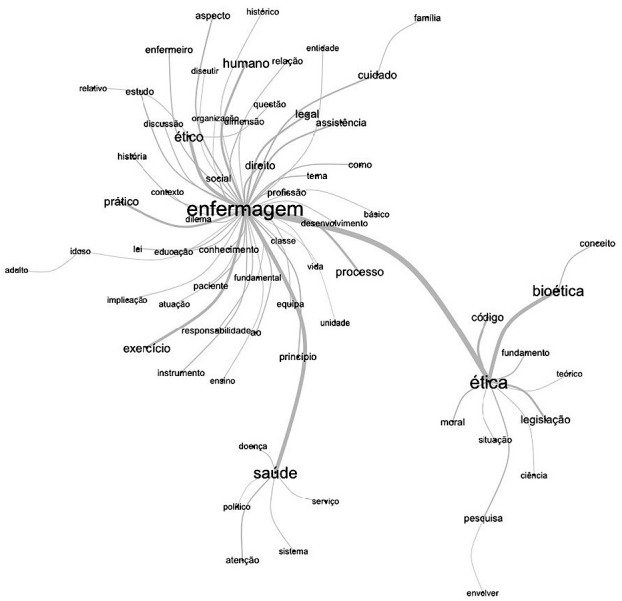
Similarity of words present in the menus of the transversal disciplines that address ethics, Brazil, 2022

The menus of the PPPs contributed to the elaboration of the corpus submitted to the similarity analysis through the IRaMuTeQ software. Such analysis, elucidated by the maximum tree, establishes a thematic structural configuration, of relative importance, which favors the understanding of the predominant relationship between the words present and establishes a hierarchical relationship between them, as shown in Figure 1.

The analysis performed by IRaMuTeQ of the words constituting the menus of the specific disciplines (corpus) was carried out in units of analysis with a frequency greater than 30. The similarity analysis presented in Figure 1 brings the three main units - namely nursing, ethics and health - that are associated in the strongest hierarchical relationships, with nursing as the central axis in this association.

The first major association is based on the word “nursing” with its dependent analysis units that indicate the professional “exercise” in the dimensions of “teaching”, “assistance” and “organizational”, with a “historical”, “legal” and “social” basis in the relationship of “care” with the “patient” and “family”.

This connection is associated with “health”, which presents a configuration of “attention” to health at different levels. The “health” connection adds few elements, which complement each other structurally, namely, the political sphere, the “system”, the “services” and “attention” to health and its impact on the “health”-“disease” process.

The second connection of “nursing” occurs with “ethics”, with a stronger connection with the “concept” of “bioethics” and, in the background, with its “theoretical” “foundation”, “code”, “moral” and “legislation”.

**Figure 2:**
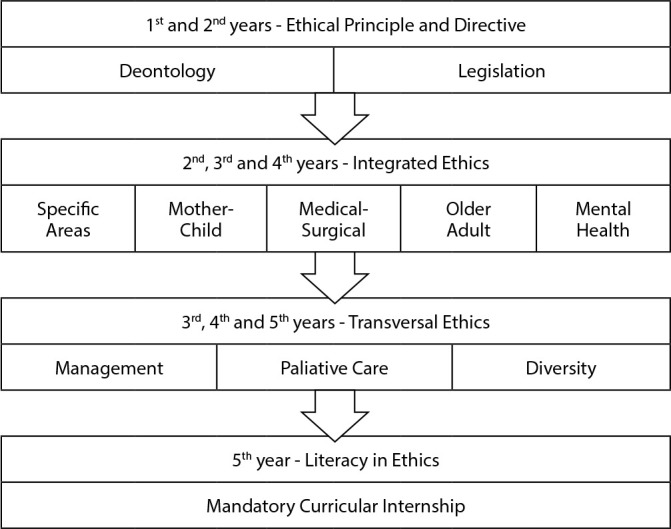
Overview of the teaching of ethics in nursing in public institutions, Brazil, 2022

When considering the findings in the PPPs analyzed, the panorama of ethics teaching in undergraduate Nursing programs in public institutions was represented in Figure 2.

According to the findings, Figure 2 shows the teaching of ethics in the first and second years of Nursing programs, focusing on deontological and legal aspects. During the program, with the introduction of specific disciplines, Ethics begins to be treated in the context of these specificities by area of clinical practice of nurses. In the final years of the program, it begins to be identified in situations transversal to clinics and becomes related to themes related to diversity, palliative care and management, for example. In the last year of the program, in the practice of the curricular internship, the emphasis given was noted so that all the knowledge acquired in relation to ethics is consolidated in the training of nurses in skills and attitudes, which is characterized as a training in literacy in ethics.

## DISCUSSION

Throughout the history of nursing, Ethics has always been presented as a reference in the configuration of its identity and professional practice. It is perceived as a principle and guideline for health promotional practice with a focus on the integrality of the being. Considering it as a principle, in the hermeneutical sense, establishes not only a beginning, but also a continuous value that underlies decisions and actions. As a principle, it becomes an assumption of the practice of care. This concept is complemented by Ethics as a guideline, since it starts to be understood as the only meaning for the movement^(15)^.

In this way, ethics is introduced in the National Nursing Curriculum Guidelines as a necessary and essential dimension in professional training. It is articulated with everyday issues so that this training guarantees to the graduates attitudes and values of citizenship, action and critical-reflective reasoning, assistance characterized by the bond and competence to solve problems inherent to the complexity of health work^(16-17)^.

Based on these considerations, the identification of the discipline of ethics at the beginning of nursing education is of great importance for the understanding of professional identity and intrinsic values in nursing care practice. Regardless of the area of activity, ethics is the foundation of professional nursing activity: whether in the administrative, assistance, educational or investigative dimension, it is expressed in relationships with the individual, family and community in organizational and interpersonal situations^(18)^.

The teaching of ethics in the first years of the program allows Nursing students to understand their social responsibility, strengthen citizenship in established relationships and favor the micropolitics of the humanized and user-centered work process. Thus, ethical guidance enhances social, political and pedagogical formation and contributes to critical reasoning in the daily practice of nursing. The formation assumes a cognitive and attitudinal character preparatory to a globalized society and with a collective thinking in constant transformation^(19)^.

It was evidenced that the teaching of ethics in the first years of undergraduate Nursing programs focuses on deontological issues. Legislation pertinent to nursing practice refers to an objective approach to ethics, fundamental for those who do not know the profession and its legal frameworks and limits. Ethics establishes the rights, duties and prohibitions of nursing in its relationship with itself, with the user and other workers and organizations. Thus, ethics is presented in a spiral beginning with professional identity, expanding in the various relationships established in professional practice and ending with the penalties applied as a last resort for the resolution of ethical problems^(20)^.

The training path of nurses after the first and second year expands the scope of professional practice by presenting specialties of nursing care. At this point, the student begins to be confronted with specific realities of some specialized activities. Thus, the teaching of ethics is configured in a process of critical-reflective reasoning integrated into the specific areas of care, with emphasis on the health of women and children, health of the older, mental health, associating with contemporary themes with permanent debate in thesocial context^(21)^.

The integrated approach to specific areas recognizes the deontological field as the foundation of ethical professional practice, but expands the ethical discussion to specific contemporary themes necessary for the training of nurses in a globalized society. Therefore, the professional training of nurses plays an important role in arousing the interest of students in relation to professional values and facilitating their incorporation into professional practice^(19)^.

The outcome of nursing education leads ethical discussions to cross-cutting themes that are manifested in different clinical and performance areas. Diversity, immigration, moral and sexual harassment, palliative care, social identity, among others, are characterized as contemporary issues that bring specific situations to the health system, with which nursing professionals are often not prepared to deal in their daily lives.

The teaching of ethics in a transversal way to the lines of care with an approach to contemporary social issues leads to a critical-reflexive training that favors the identification of an expanded health-disease process in its conception, since it perceives the individual in his/her expanded health condition and related to all its determinants. The conception of comprehensive care becomes the result of a systemic view of the factors that interfere with the quality of life and decrease the functional capacity of the citizen. Integrated and contemporary ethics perceives the relationship with nature, citizenship, inclusion and social responsibility as interfering elements in the health disease process and expands the concept of care beyond the specific clinical issues of nursing care^(22-24)^.

Integrated and transversal ethics enables the reflection of being and practicing nursing in a society characterized by demographic, epidemiological and care transition and recognizes the influence of determining and conditioning factors of health, such as social, cultural, religious and commercial factors^(25-27)^.

In this sense, ethics becomes an object of complex and dynamic study and, consequently, needs to be understood as a living organism by students in the translation of ethical principles and precepts for clinical practice in the real context. Ethics teaching needs to take place in an environment where new professional roles can be observed and practiced. In this sense, it is evident the responsibility of educators to integrate the theoretical aspects of ethics with the clinical experiences of students^(23-24,27)^.

In the training process of nurses, this integration between theory and practice occurs fully and conclusively in the Mandatory Curricular Internship. The ethical attitude expected from Nursing students in their training process can be verified in this phase of training, since the student is involved with professional practice throughout the academic period and deals with all issues related to care in their decision-making process.

The curricular internship is presented as an opportunity to validate technical and ethical training. When considering the concept of Health Literacy, which emphasizes the cognitive and social skills of an individual related to the access, understanding and use of health information to protect and promote health^(28)^, it is also possible to attribute this concept to ethical training and to attribute, as an expectation of training of Nursing students, a double literacy: Health Literacy and Ethical Literacy.

Thus, this study findings point to a parallel between the training process in nursing and the professional’s ethical training cycle. The concept of ethics, even if initially treated in an isolated and legalistic way, must be gradually incorporated into specific activities and correlated to the multiple variables imposed by the diversity of modern society. In addition, it was also evident the need for the training of nurses to guarantee both the acquisition of knowledge by a critical-reflective process and their attitudinal appropriation to be consolidated in the Mandatory Curricular Internship.

### Study limitations

The main limitation of the study was the unavailability of the pedagogical projects of the nursing programs on the websites of the universities or programs. Almost 30% of the programs did not publish their projects, being a significant representation for the national panorama in the teaching of ethics.

### Contributions to the field

This study presents how the teaching of nursing ethics has been carried out in public universities in Brazil. Capturing this picture contributes directly to the discussions on how the ethical training of nursing in the Brazilian scenario is oriented and collaborates with the programs under development and/or in updating their pedagogical projects.

## FINAL CONSIDERATIONS

This study findings show that Ethics is a consolidated theme in nursing training, present in all pedagogical projects available for consultation. It is clear that deontological teaching is the foundation and determinant for the entire formative process. It provides the basis for establishing the legal and ethical parameters for any and all professional practice in their interpersonal, intraprofessional, interprofessional and inter-institutional relationship.

In addition to this ethical training as a principle and guideline, this study points to the importance of ethics teaching being perceived in the context of specific nursing activities, that is, that ethics be defined and applied in the daily care, in the various specialties where nursing offers its care. Nursing education should begin to elaborate a teaching-learning process in which the undergraduate nursing students can identify, discuss and articulate a new language of the ethical assumption of nursing specific to each discipline of their training.

The great challenge in teaching ethics in undergraduate nursing programs is its integration with the reality experienced by nursing to the point of strengthening the idea that ethics is performed daily, in each action of caring, teaching and managing, as an assumption in critical-reflective reasoning and in each decision-making.
